# Inflammatory Fibroid Polyp (Vanek’s Tumor): A Retrospective Multicentric Analysis of 67 Cases

**DOI:** 10.3390/cancers17071209

**Published:** 2025-04-02

**Authors:** Mario Martín Sánchez, Víctor Domínguez-Prieto, Siyuan Qian Zhang, Hernán Darío Quiceno Arias, María Bernarda Álvarez Álvarez, Montiel Jiménez Fuertes, Cecilia Meliga, Santos Jiménez-Galanes, Héctor Guadalajara Labajo, Damián García Olmo, Pedro Villarejo Campos

**Affiliations:** 1 Department of Surgery, Fundación Jiménez Díaz University Hospital, Avenida de los Reyes Católicos 2, 28040 Madrid, Spainvictor.dominguez@quironsalud.es (V.D.-P.);; 2 Department of Pathology, Fundación Jiménez Díaz University Hospital, Avenida de los Reyes Católicos 2, 28040 Madrid, Spain; 3 Department of Surgery, University Hospital Infanta Elena, 28342 Madrid, Spain; 4 Department of Surgery, Universidad Autónoma de Madrid, 28049 Madrid, Spain

**Keywords:** Vanek’s tumor, inflammatory fibroid polyp, intussusception, gastrointestinal submucosal tumor, endoscopy, surgery

## Abstract

Objectives: Inflammatory fibroid polyps, also known as Vanek’s tumors, are rare benign lesions of the gastrointestinal tract. Although the exact cause remains unclear, several theories suggest an association with inflammatory processes and genetic factors. This study aims to present the largest cohort of inflammatory fibroid polyp cases to date, analyzing their clinical presentation, diagnostic methods, and treatment approaches. Materials and methods: A retrospective multicentric analysis was conducted on 67 patients diagnosed with inflammatory fibroid polyps between 2013 and 2023 across four hospitals. Clinical data regarding tumor location, size, symptoms, and treatment were collected. Non-parametric statistical tests, including the chi-square test, Cramér’s V coefficient, and the Mann–Whitney U test, were used to identify association between tumor characteristics, location, and treatment outcomes. Results: The cohort included 67 patients (58.2% female, median age 60 years). The stomach was the most common tumor site (47.8%), followed by the colon (32.8%), and small intestine (10.4%). The majority of patients (73.1%) were asymptomatic, while 9% experienced intestinal obstruction, all of which were located in the small intestine. Endoscopic resection was successful in 77.6% of cases, but surgical intervention was more frequently required for tumors in the small intestine. A significant association was found between larger tumor size, emergency presentation, intestinal location, and the need for surgery. Conclusions: Inflammatory fibroid polyps are commonly managed with endoscopic resection, particularly in gastric and colonic locations. However, small intestinal tumors more often need surgical treatment, especially when presenting with complications like intestinal obstruction.

## Simple Summary

Inflammatory fibroid polyps, also known as Vanek’s tumors, are rare benign lesions of the gastrointestinal tract that can mimic malignant conditions, posing a diagnostic challenge. This study presents the largest multicentric analysis of these tumors to date, aiming to characterize their clinical presentation, anatomical distribution, and management strategies. We provide valuable insights into the effectiveness of endoscopic and surgical approaches, particularly in relation to tumor location and size. Our findings may contribute to optimizing diagnostic accuracy, guiding treatment decisions, and reducing unnecessary surgical interventions.

## 1. Introduction

Inflammatory fibroid polyp (IFP), also known as Vanek’s tumor, was first documented by J. Vanek in 1949. This publication included six patients with diverse clinical presentations in whom he identified a peculiar submucosal gastric lesion described as a “submucosal granuloma with an eosinophilic infiltration” [1]. These tumors are defined as benign, idiopathic, and localized neoplastic lesions in the submucosa of gastrointestinal (GI) tract [2].

Vanek’s tumor represents less than 0.1% of all gastric polyps [3]. Epidemiologically, IFP affects both sexes equally. It has a wide age range, with a peak of incidence in the fifth to seventh decades of life.

In the anatomopathological examination, IFPs are pedunculated or sessile lesions that vary in size, often projecting into the bowel lumen. Microscopically, these lesions are characterized by a spindle and stellate cell proliferation within a fibrous or myxoid stroma, highly vascular, and accompanied with significant inflammatory infiltrate, predominantly eosinophilic [2,4].

Immunohistochemically, Vanek’s tumors consistently express CD34 and vimentin while lacking CD117. This distinctive profile is crucial for distinguishing IFP from GIST and other mesenchymal tumors that may share similar morphological features [5,6]. At the molecular level, significant advances have identified activating mutations in the platelet-derived growth factor receptor alpha (PDGFRA) gene in up to 70% of IFPs [7,8]. However, the presence of PDGFRA mutations in both IFP and a subset of GISTs may complicate the differential diagnosis, particularly in cases where GIST lacks CD117 expression [9].

Although they can be found throughout the GI tract, IFPs are commonly located at the gastric antrum or ileum. They are typically asymptomatic. When present, clinical manifestations can vary according to size and location: abdominal pain is frequently observed with IFPs located in the stomach, while obstruction and intussusception are more characteristic when the tumor is found in the small intestine [10,11,12,13]. Colonic IFP, in contrast, may resemble symptoms of a malignant tumor such as iron deficiency anemia, hematochezia, or change in bowel habits [14,15].

Radiological imaging such as ultrasonography, computer tomography (CT) or magnetic resonance imaging (MRI) are commonly used for diagnosis of IFP’s complications. Regarding treatment, most IFPs are diagnosed and treated by endoscopic resection [16,17,18]. Surgical intervention is reserved for cases where endoscopic resection is not feasible due to tumor size or location. Furthermore, sometimes urgent surgery is needed primarily in case of intussusception or obstruction [19,20,21].

Most available studies on IFP are based on small retrospectives series, limiting the ability to draw definitive conclusions regarding their clinical behavior and management [22,23].

To our knowledge, this study represents the largest cohort of patients with Vanek’s tumor reported to date. By documenting our findings, we aim to provide a comprehensive analysis of IFP by investigating its clinical presentation, diagnostic modalities, and treatment options. This study seeks to enhance the understanding of this rare entity and contribute to establishing more consistent diagnostic and therapeutic strategies.

## 2. Materials and Methods

A multicenter retrospective analysis was conducted on all patients diagnosed histologically with IFP or Vanek’s tumor from 2013 to 2023 from four hospitals in Madrid (Spain): Hospital Universitario Fundación Jiménez Díaz, Hospital Universitario Rey Juan Carlos, Hospital Universitario General de Villalba, and Hospital Universitario Infanta Elena. The participating centers included one tertiary referral hospital (Hospital Universitario Fundación Jiménez Díaz) and three secondary-level hospitals, collectively serving a population of approximately 850,000 inhabitants within the Madrid region.

### 2.1. Patient Selection and Data Collection

Patients were identified through a comprehensive review of pathological databases across the participating institutions. Most diagnoses were established incidentally through endoscopic procedures, while others were obtained from surgical specimens. Currently, there are no universally standardized diagnosis criteria for IFP. However, all cases were analyzed by experienced pathologists following histopathological criteria to ensure diagnostic accuracy and consistency across centers.

All participating centers had access to the same diagnostic and therapeutic resources, ensuring a uniform approach to patient management. In the absence of standardized guidelines, treatment decisions were based on clinical judgment and institutional protocols, which were comparable across all hospitals.

Clinical data were retrospectively collected from electronic medical records and institutional databases. Patients were identified based on histopathological reports confirming IFPs. The information gathered included patient demographics (age and sex), tumor characteristics (size, location, histological findings), clinical presentation, diagnosis, treatment approach, and follow-up. Exclusion criteria included cases with incomplete medical records or cases with diagnostic uncertainty after histological examination.

### 2.2. Statistical Analysis

Initially, the Kolmogorov–Smirnov (KS) test was applied to assess the normality distribution of all variables. In this study, all variables resulted in a KS test *p* < 0.05, leading to the application of non-parametric tests.

The Kruskal–Wallis test was used to compare the relationship between the categorical and the continuous variables. The chi-square ($\chi^{2}$) test was employed to compare two qualitative variables. Therefore, Cramér’s V coefficient was applied to evaluate the strength of the association, with values closer to 1 indicating a stronger association. The Mann–Whitney U test was utilized as a non-parametric test to analyze relationships between quantitative and qualitative variables. Additionally, Cohen’s R was used to quantify the effect size in this comparison. Statistical significance was set at *p* < 0.05.

The statistical analyses were performed using SPSS software, version 25.

## 3. Results

A total of 67 patients were included, consisting of 39 females (58.2%) and 28 males (41.8%), with a median age of 60 years (range 32–83 years).

### 3.1. Tumor Location

Regarding tumor location (Table 1), the stomach was the main location, involving 32 patients (47.8%). Vanek’s tumor was diagnosed in 22 patients (32.8%) in the large intestine, while 7 patients (10.4%) had the tumor located in the small intestine. Additionally, the rectum was affected in 5 patients (7.5%), and the esophagus in 1 patient (1.5%).

The most common site in the stomach was the antrum, observed in 25 patients (78.1% of gastric IFPs). The pylorus and corpus were affected in 4 and 3 patients, respectively (12.5% and 9.4% of gastric IFPs). Regarding colonic IFP, 17 patients (77.3% of colonic IFPs) had IFP localized in the left colon, 3 patients (13.6% of colonic IFP) in the right colon and 2 patients (9.1% of colonic IFPs) in the transverse colon.

In relation to tumor location and demographic characteristics (Table 2), among male patients, 12 patients (42.9%) had gastric IFPs, 9 patients (32.1%) had colonic IFPs, 4 patients (14.3%) had rectal IFPs, and 2 patients (7.1%) had small intestine lesions. In contrast, among female patients, 20 patients (51.3%) had gastric IFPs, 13 patients (33.3%) had colonic IFPs, 5 patients (12.8%) had small intestine lesions, and 1 patient (2.6%) had a rectal lesion. The only esophageal IFP was observed in a male patient. There was no statistically significant association between tumor location and sex (*p* = 0.282).

The mean age of the patients varied across different tumor locations. The highest mean age was observed in patients with tumors located in the stomach (63 years), followed by those with tumors in the colon (59 years). Patients with esophageal tumors had a mean age of 57 years, while those with tumors in the rectum (56 years) and small intestine (55 years) had the lowest mean ages. However, no statistically significant relationship was found between age and tumor location (*p* = 0.488).

### 3.2. Clinical Presentation

The majority of diagnoses were incidental, with 49 patients (73.1%) being asymptomatic. Among them, 24 cases (35.8%) had gastric IFPs, 18 (27%) had colonic IFPs, 5 (7.5%) had rectal IFPs, 1 (1.5%) had a small intestinal IFP, and 1 (1.5%) had an esophageal IFP. Six patients (9%) presented to the emergency department with intestinal obstruction, all of whom had small intestinal IFPs. Three patients (4.5%) were diagnosed due to iron deficiency anemia, exclusively in those with gastric lesions. Upper gastrointestinal bleeding was observed in one patient (1.5%) with a gastric IFP, while lower gastrointestinal bleeding occurred in one patient (1.5%) with a colonic IFP. Additionally, nine patients (13.4%) reported nonspecific gastrointestinal symptoms, including dyspepsia, diarrhea, or early satiety, affecting five patients (7.5%) with gastric IFPs, two patients (3%) with colonic IFPs, one patient (1.5%) with a small intestinal IFP, and one patient (1.5%) with a rectal IFP. The relationship between symptoms and tumor location is detailed in Table 3.

Among the 32 patients with gastric IFP, 29 patients had chronic gastritis in the histological examination (87.9%) and *Helicobacter pylori* was present in 6 patients (18.2%). In line with the hypothesis of an inflammatory origin, no signs of macroscopic or microscopic chronic inflammation were observed in IFP localized in esophagus or rectum. Regarding colonic IFP, only one case exhibited microscopic evidence of indeterminate chronic inflammation.

The median tumor size was 1.67 cm (range 0.2–14 cm). While most inflammatory fibroid polyps are small, one case in our study involved a tumor reaching 14 cm in the colon. The IFP in the esophagus and rectum measured 0.6 cm, while those in the stomach and colon had a median size of 1.6 cm. In contrast, tumors in the small intestine were generally larger, with a median of 3.4 cm. All patients had a single tumor except for one (1.5%), who required a second surgery due the presence of another suspicious tumor in the small intestine.

### 3.3. Diagnostic Methods and Treatment

The diagnosis was primarily established through endoscopy, accounting for 57 cases (85.1%). CT was employed in seven patients (10.4%) and diagnosis was confirmed postoperatively in three cases (4.5%). Emergency diagnosis was defined as cases in which patients presented to the emergency department with acute symptoms directly related to the tumor, leading to its identification. A total of six patients (9%) were diagnosed in an emergency setting, all of whom presented with intestinal obstruction due to small bowel IFPs.

Vanek’s tumors were successfully resected endoscopically in 52 patients (77.6%), whereas surgical intervention was necessary for 15 patients (22.4%). Among those who underwent endoscopic resection, 26 patients (50%) had gastric IFP, 20 (38.4%) had colonic IFP, 5 (9.6%) in the rectum, and 1 patient (2%) had an esophageal lesion. In contrast, surgical treatment was performed in seven cases (46.7%) with IFP localized in small intestine, six in the stomach (40%), and two in the colon (13.3%). Urgent surgery was required in six patients (40% of all surgical cases), all of whom had tumors located in the small intestine. Notably, intestinal obstruction was the indication for surgery in these cases, accounting for 85% of all IFPs found in the small intestine. Clear resection margins were obtained in all cases, ensuring complete tumor removal. All data are summarized in Table 4.

### 3.4. Follow-Up and Recurrence

The median follow-up was 68.3 months (range from 11 to 136 months), and no recurrence was observed in any case. Additional information about patients and tumor characteristics are outlined in Table 5.

### 3.5. Statistical Analysis

#### 3.5.1. Association Between Tumor Location and Treatment

A statistically significant association (*p* < 0.05) between tumor location and treatment was observed, with Cramér’s V = 0.65 indicating a strong association. While Vanek’s tumor in the stomach or colon is often managed by endoscopic resection, small intestine IFPs are more prone to need a surgical procedure.

#### 3.5.2. Association Between Tumor Size and Treatment

Regarding tumor size and treatment, a significant association is also observed (*p* < 0.05). Larger tumor size correlates with an increased probability of requiring surgery, with a moderate effect size (Cohen’s r = 0.47).

#### 3.5.3. Association Between Diagnosis and Treatment

Concerning emergency diagnosis, a significant association (*p* < 0.05) is found with the need of surgical treatment, demonstrating a moderate association (Cramér’s V = 0.459). Additionally, there is a strong association (Cramér’s V = 0.81) between emergency diagnosis and clinical manifestations.

#### 3.5.4. Association Between Tumor Location and Diagnosis

Finally, a significant and a strong association (Cramér’s V = 0.75) is found between emergency diagnosis and location (*p* < 0.05). The small intestine is the most frequent location in the emergency setting; it is usually associated with intestinal obstruction due to intussusception.

All statistical associations and their corresponding *p*-values are summarized in Table 6.

## 4. Discussion

To our knowledge, this study includes the largest cohort of IFP cases reported in the scientific literature to date, with a total of 67 patients. Our results are mainly consistent with previous studies regarding location, symptoms, diagnosis, and treatment. Additionally, we have performed a detailed statistical analysis that offers a better understanding of this rare entity [4,22,24].

Our study concurs that Vanek’s tumor predominantly affects individuals in the fifth to seventh decades of life, with a median age of 60 years. There is a slight female predominance, according to our results. This is congruent with the epidemiological data reported in the most recent literature reviews [4,22,23,24,25].

Although etiology remains uncertain, different theories have been proposed: a reactive inflammatory response (associated mainly with *Helicobacter pylori*), an allergic reaction, or an autoimmune process [2,4]. In addition, some cases have been associated with Crohn’s disease, suggesting a potential association with inflammatory bowel conditions [26,27,28]. Chronic gastritis was observed in nearly 90% of cases, supporting the hypothesis that Vanek’s tumor may arise as a result of a reactive inflammatory process [2,4,29]. In non-gastric locations, we did not identify signs of chronic inflammation in the surrounding tissue, with the exception of one case in the colon. *H. pylori* was present in only 18% of cases, which is substantially lower than the general prevalence of *H. pylori* infection in Spain, which is approximately 40%, according to a seroprevalence study [30]. This disparity suggests that *H. pylori* infection may not represent a significant risk factor for IFP development.

The most common location for IFP is the stomach. Although other articles report the small intestine as the second most frequent site, our study indicates that the colon is the second most frequent location [4]. This discrepancy may be explained by the high frequency of colorectal cancer screening conducted in our environment, as well as the fact that most relevant literature reviews are focused on IFP causing intestinal intussusception [23,24].

In terms of clinical presentation, our data underscore the variable symptomatology of IFP. In contrast to other studies where pain is the most common symptom, our findings demonstrate that most patients are asymptomatic [4,24]. Screening endoscopies could also explain this difference. However, when symptoms do occur, nonspecific gastrointestinal symptoms and intestinal obstruction are the most prevalent, whereas gastrointestinal bleeding remains rare. Additionally, a strong association was identified between emergency diagnoses, clinical manifestations, and tumor location. This correlation confirms that patients presenting in urgent care settings exhibit a substantially higher likelihood of manifesting obstructive symptoms (predominantly resulting from intussusception).

Regarding treatment, the majority of IFPs in our study were successfully managed endoscopically (77.6%), particularly in gastric and colonic locations. Other studies report a wide range of percentages for endoscopic resection which vary from 20 to 60% [4,22,25]. Based on our data, gastric tumors are often successfully managed with endoscopic procedures, whereas small intestine tumors are more inclined to require surgical intervention due to intestinal obstruction. In fact, 85% of IFPs located in the small intestine needed urgent surgery. Advances in endoscopic techniques such as endoscopic mucosal resection (EMR) have broadened the range of lesions amenable to non-surgical removal. EMR is generally reserved for cases in which standard snare polypectomy may be insufficient, such as larger lesions or sessile polyps, where achieving complete resection requires a more advanced technique [18,31,32].

The primary challenge associated with small intestine involvement is the difficulty in achieving an early diagnosis due to the low precision of CT and the limited accessibility of this region to endoscopy. Consequently, these tumors often remain undetected until patients develop symptoms.

Our analysis further suggests that larger tumor size and emergency diagnosis have a moderate association with the requirement of surgical intervention. Indeed, all patients who presented in the emergency department needed an urgent surgery. In case of intestinal intussusception, management can be approached either by *en bloc* resection or reduction followed by resection. *En bloc* resection is strongly recommended in case of ileo-colic intussusception or when there is a high suspicion of malignancy. For small intestine intussusception, reduction followed by resection may be advisable when it can be performed safely [24,33,34].

The prognosis following complete removal of IFP is excellent, with a negligible risk of recurrence based on our data and only a single case of recurrence reported in the literature. Hence, long-term follow-up is generally not required after confirmed complete excision [35].

Some limitations should be considered in this analysis of IFPs. This retrospective study is inherently limited by selection and information biases. Our analysis was restricted to patients with symptomatic presentations or incidental endoscopic findings, potentially leading to an underdiagnosis of IFP in those who did not seek medical attention or underwent screening. The multicenter nature introduces variability between centers, which may reduce the precision of results. However, this multicenter design also enhances the external validity of the findings, making them more applicable to the broader population. Despite these limitations, the study provides valuable insights into these rare polyps, contributing significantly to the understanding of their characteristics and management.

## 5. Conclusions

This study provides the largest multicentric cohort of Vanek’s tumor cases to date, reinforcing the understanding of its clinical presentation, diagnostic challenges, and treatment outcomes. Our findings highlight the efficacy of endoscopic management, especially for gastric and colonic tumors, while underscoring the need for surgical intervention in small intestine cases. Prognosis after complete resection is excellent due to its benign nature, with only one recurrence reported in the literature. These results contribute significantly to the management and decision-making of Vanek’s tumors.

## Figures and Tables

**Table 1:** Vanek’s tumor locations along the GI tract.

Location	Total	
**Stomach**	32	47.8%
Antrum	25	(78.1% of gastric tumors)
Corpus	4	(12.5% of gastric tumors)
Pylorus	3	(9.4% of gastric tumors)
**Colon**	22	32.8%
Left colon	17	(77.3% of colonic tumors)
Right colon	3	(13.6% of colonic tumors)
Transverse colon	2	(9.1% of colonic tumors)
**Small intestine**	7	10.4%
**Rectum**	5	7.5%
**Esophagus**	1	1.5%

**Table 2:** Distribution of tumor locations by sex and mean age.

Location	Male	Female	Mean Age
Esophagus	1 (3.6%)	0 (0%)	57
Stomach	12 (42.9%)	20 (51.3%)	63
Small intestine	2 (7.1%)	5 (12.8%)	55
Colon	9 (32.1%)	13 (33.3%)	59
Rectum	4 (14.3%)	1 (2.6%)	56
Total	28 (100%)	39 (100%)	

**Table 3:** Locations and clinical manifestations of IFP.

	Esophagus	Stomach	Small Intestine	Colon	Rectum
Asymptomatic	1 (1.5%)	24 (35.8%)	1 (1.5%)	18 (27%)	5 (7.5%)
Upper GI bleeding	0 (0%)	1 (1.5%)	0 (0%)	0 (0%)	0 (0%)
Lower GI bleeding	0 (0%)	0 (0%)	0 (0%)	2 (3%)	0 (0%)
Anemia	0 (0%)	3 (4.5%)	0 (0%)	0 (0%)	0 (0%)
Intestinal obstruction	0 (0%)	0 (0%)	6 (9%)	0 (0%)	0 (0%)
Nonspecific symptoms	0 (0%)	4 (6%)	0 (0%)	2 (3%)	0 (0%)

**Table 4:** Locations and treatment of IFP.

Location	Endoscopy Resection	Surgical Procedure
Esophagus	1/1 (100%)	0/1 (0%)
Stomach	26/32 (81.3%)	6/32 (18.7%)
Small intestine	0/7 (0%)	7/7 (100%)
Colon	20/22 (90.9%)	2/22 (9.1%)
Rectum	5/5 (100%)	0/5 (0%)

**Table 5:** Patients and tumor characteristics.

Variable	Total (*n* = 67)
Age (years)	60 (32–83)
Sex	Female: 39 (58.2%) Male: 28 (41.8%)
Clinical manifestations	Asymptomatic: 49 (73.1%) Upper GI bleeding: 1 (1.5%) Lower GI bleeding: 2 (3%) Anemia: 3 (4.5%) Obstruction: 6 (9%) Nonspecific symptoms: 6 (9%)
Helicobacter pylori	6 (18.2% of gastric tumor)
Chronic gastritis	29 (87.9% of gastric tumor)
Unique tumor	66 (98.5%)
Tumor size (cm)	1.67 (range 0.2–14)
Diagnosis	Endoscopy: 57 (85.1%) CT: 7 (10.4%) Surgery: 3 (4.5%)
Emergency diagnosis	6 (9%)
Treatment	Endoscopic resection: 52 (77.4%). Surgical treatment: 15 (22.4%)
Need of urgent treatment	6 (9%)
Recurrence	0 (0%)
Follow-up time (months)	68.3 (11–136)

**Table 6:** Statistical associations.

Associations	*p*-Value
Tumor location and sex	*p* = 0.282
Tumor location and age	*p* = 0.488
Tumor location and treatment	*p* < 0.05
Tumor size and treatment	*p* < 0.05
Diagnosis and treatment	*p* < 0.05
Tumor location and diagnosis	*p* < 0.05

## Data Availability

The data presented in this study is available on request from the corresponding author. The data are not publicly available due to ethical restrictions.

## Abbreviations

The following abbreviations are used in this manuscript:

CT	Computer Tomography
EMR	Endoscopic Mucosal Resection
GI	Gastrointestinal
GIST	Gastrointestinal Stromal Tumor
IFP	Inflammatory fibroid polyp
KS	Kolmogorov–Smirnov
MRI	Magnetic Resonance Imaging
PDGFRA	Platelet-Derived Growth Factor Receptor Alpha
